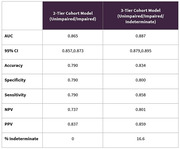# Digital Cognitive Screening and ML‐Enabled Random Forest Modeling for the Detection of Cognitive Impairment

**DOI:** 10.1002/alz.089936

**Published:** 2025-01-09

**Authors:** Russell Banks, Karl Thompson, Ali Jannati, Sean Tobyne, John Showalter, David Bates, Alvaro Pascual‐Leone

**Affiliations:** ^1^ Linus Health, Boston, MA USA; ^2^ Michigan State University, East Landing, MI USA; ^3^ Harvard Medical School, Boston, MA USA; ^4^ Boston Children's Hospital, Harvard Medical School, Boston, MA USA; ^5^ Hinda and Arthur Marcus Institute for Aging Research and Deanna and Sidney Wolk Center for Memory Health, Hebrew SeniorLife, Boston, MA USA; ^6^ Linus Health, Waltham, MA USA; ^7^ Hinda and Arthur Marcus Institute for Aging Research at Hebrew SeniorLife, Boston, MA USA; ^8^ Guttmann Brain Health Institute, Institut Guttmann, Institut Universitari de Neurorehabilitació Adscrit a la UAB., Badalona, Barcelona Spain; ^9^ Department of Neurology, Harvard Medical School, Boston, MA USA

## Abstract

**Background:**

Mild cognitive impairment (MCI), is characterized by cognitive dysfunction not severe enough to affect one’s activities of daily living (ADLs)1. Annually, approximately 15‐20% adults 65 and older will present with MCI 1. MCI is considered a significant risk factor and a robust predictor for developing dementia. The time course for progression to dementia can vary substantially between individuals and is impacted by the specific pathology underlying the MCI, and the cognitive deficits associated with cognitive impairment (CI) subtypes 2,3. Despite the conversion risk of MCI to dementia and the effectiveness of early lifestyle interventions to mitigate the conversion risk, many investigations do not account for MCI in their CI prediction models. This research investigates binary and 3‐class ML‐enabled modeling to classify CI status leveraging multiple modalities of cognition extracted from the Digital Clock and Recall (DCR), a brief digital cognitive assessment.

**Method:**

Data from 983 participants in the Bio‐Hermes‐001 multi‐site study (age mean±SD=72±6.7; 56% female; years of education mean±SD=15±2.7; primary language English), a priori classified as cognitively unimpaired (CU; n=417), mild cognitively impaired (n=309), or probable Alzheimer’s dementia (n=257) based on expert consensus clinical diagnosis and neuropsychological evaluation were analyzed. A random forest model was trained on DCTclock and word recall data to classify cognitive impairment using a binary (CI and CU) and 3‐tier (CI, Indeterminate, and CU) prediction thresholding schemes.

**Result:**

The 3‐tier model predictions performed well (AUC=0.887; accuracy=0.834; NPV=0.801; PPV=0.859) outperforming the binary predictions (AUC=0.865; accuracy=0.79; NPV=0.737; PPV=0.837) when measured on Biohermes’ cohort diagnosis. The sensitivity and specificity of the 3‐tier predictions were 0.858 and 0.8, respectively.

**Conclusion:**

The DCR, a 3‐minute digital cognitive assessment can be used to classify MCI and probable Alzheimer’s dementia with high accuracy, NPV, and PPV.